# Self-management support from health care providers in Shared Medical Appointments: Didactic techniques, peer learning, group dynamics and motivation

**DOI:** 10.1016/j.pecinn.2024.100337

**Published:** 2024-08-23

**Authors:** Anna H.C. Tsiamparlis-Wildeboer, Esther I. Feijen-De Jong, Elke Tichelman, Ank de Jonge, Fedde Scheele

**Affiliations:** a Department of Primary and Long-Term Care, University of Groningen, Groningen, the Netherlands; b Midwifery Academy Amsterdam Groningen, Inholland, Groningen, the Netherlands; c Midwifery Science, Amsterdam UMC, Amsterdam, the Netherlands; d Amsterdam Public Health, Quality of Care, Amsterdam, the Netherlands; e Department of Midwifery Education, Rotterdam University of Applied Sciences, Rotterdam, the Netherlands; f Research Centre Innovations in Care, Rotterdam University of Applied Sciences, Rotterdam, the Netherlands; g Amsterdam Reproduction and Development, Amsterdam, the Netherlands; h Athena Institute for Transdisciplinary Research, Vrije Universiteit Amsterdam, Amsterdam, the Netherlands; i Amsterdam UMC, Amsterdam, the Netherlands

**Keywords:** Shared Medical Appointments, Self-management support, Health care providers, Peer learning, Group dynamics, Motivation

## Abstract

**Objective:**

We investigated the support of self-management by health care providers (HCP) in prenatal Shared Medical Appointments (SMA).

**Methods:**

on an topic list, semi-structured interviews were conducted. HCP who provided prenatal care in SMA in the last five years were recruited. Thematic analysis was used.

**Results:**

We conducted 15 interviews. Four research themes were defined: didactic techniques, peer learning, motivation and the health care providers. Self-management support in SMA is based on peer-learning and is influenced by group dynamics. HCP play a role in the creation of an effective learning climate by using practical and communication techniques. HCP motivate participants for self-management through peer learning and person centered care. HCP need certain personality traits and leadership skills.

**Conclusion:**

Self-management support in SMA is based on peer-learning and is influenced by group dynamics. HCP create an effective learning climate using practical and communication techniques and motivate participants for self-management through peer learning and person-centered care.

**Innovation:**

This is the first study that gives insight in self-management support in SMA. HCP and medical schools should be aware of the fact that HCP in SMA need insight in didactic techniques, peer learning, group dynamics and leadership skills.

## Highlights

• Self-management support in SMA is based on **peer learning.**
• Self-management support is influenced by **group dynamics.**
• HCP contribute to an **effective learning climate** through **practical** and **communication techniques.**
• HCP **motivate** for self-management through **peer learning** and **person centered care.**
• HCP in SMA need **personality traits** and **leadership skills.**

## 1 Introduction

Self-management support is an important cornerstone of Shared Medical Appointments (SMA), but how do health care providers (HCP) manage to support self-management in a group and what is the influence of group dynamics? According to Huber et al., health is not the absence of illness, but “the ability to adapt and to self-manage, in the face of social, physical and emotional challenges” [1]. This new dynamic concept of health requires from patients to obtain self-management skills such as developing coping strategies and increasing self-efficacy [2,3]. By providing effective educational interventions for self-management, HCP can empower individuals to acquire these self-management skills [[2], [3], [4]]. In this way, HCP contribute to better health outcomes and improved quality of life [5,6].

Key components of effective educational strategies are person-centered care [2,[7], [8], [9]], the tailoring of self-management support to the individuals' capacities and needs [2,8,10] and motivational support [[10], [11], [12]]. For HCP, person-centered care means focusing on what is important to the individual, offering choices [2] and providing holistic care [2,7,8,11,[13], [14], [15], [16], [17], [18]]. Important aspects of tailored self-management support are the use of different communication styles [8] and being aware of individuals' health literacy. [[7], [8], [9],^12^,^14^,[19], [20], [21]]

Person-centered care and tailored self-management support can influence the awareness of individuals for self-management, however, for the adoption of self-management skills, individuals need motivation [2,10,11]. According to the I-Change model for behavioral changes [22], motivation consists of three components, namely attitude, social influences and self-efficacy. HCP can contribute to motivation in helping individuals to set goals [8,9,23], to make action plans [9,23] and in raising their self-confidence [24]. Proved techniques for maximizing motivation in self-support management are motivational interviewing [2,3,10,25], positive stimulation and regular evaluation [[26], [27], [28]]. Social influences from partners and family members also play a role in motivation [2,13,17,23].

Studies about patient education for type 2 diabetic patients showed that group-based educational interventions are more effective than individual education at obtaining self-management skills [[29], [30], [31]]. A valuable method for group-based self-management education is the use of Shared Medical Appointments [32]. Under the name CenteringPregnancy©, prenatal SMA are quite common in the Netherlands. In these SMA in hospitals and community settings, 8 to 12 pregnant women with approximately the same gestational age gather in nine appointments for prenatal care and education. SMA are led by two HCP (at least one of them is a midwife, the other can be a midwife, physician assistant, maternity care assistant, doctor's assistant, student etc.) and they last 120 min, replacing the individual appointments in prenatal care. In these SMA, the HCP give one-on-one time to the participants by doing physical check-ups, but they also provide group-based patient education aiming at the support of self-management [33,34]. This patient education is based on a lesson guide with 17 themes (e.g. nutrition, breastfeeding), related to positive pregnancy outcomes, good parenthood and healthy life style [33,35]. The guide also contains self-assessment forms with the aim of encouraging participants to think about their own health. In order to be able to provide care in SMA, the HCP are trained in a post-graduate two-days course where they learn about the principles and structure of SMA and the use of the lesson guide. Participation in prenatal SMA is voluntary in the Netherlands, but the growing number of HCP that provides prenatal care in SMA and the growing number of participants in these SMA proves the popularity of this program.

Given the fact that the key components of educational interventions for self-management support are person-centered care, the tailoring of self-management support and motivational support, the questions arise how HCP in SMA perform group education based on these components and how this education is influenced by group dynamics. Studies under (para)medical students showed that certain elements of group dynamics (e.g. openness, support) promote learning [36] and that some behaviours can have positive impact on learning (e.g. sharing information, encouraging others to participate in the discussion, while other behaviours (e.g. passiveness) have negative impact [37]. However, limited research has been performed about the support of self-management in groups from the perspective of the HCP. According to Barkham & Ersser [38], HCP needed extra training in group facilitation and according to Hughes et al. [24], HCP focused on education more than on self-management skills and they paid little attention to motivation for health behaviour changes. A focus on information and awareness rather than on motivation was also found by Tsiamparlis et al. [39] In a study of Salemonsen et al. [40], HCP highlighted relational and communicational skills as essential for group self-management support. However, to our knowledge, no study has been performed about the techniques the HCP use when they support self-management and how they motivate participants to adopt self-management skills neither did we find any study about the influence of group dynamics on this support. As we think that research about these topics could have added value for patient education, we conducted a qualitative study based on the following research questions:

1. Which techniques do health care providers in Shared Medical Appointments use in supporting self-management and how do they motivate participants for the adoption of self-management skills?
2. What is in the perception of the health care providers the influence of group dynamics on self-management support in Shared Medical Appointments and how do they deal with this?

## 2 Methods

A qualitative design based on semi-structured interviews was used for this study. Data collection was carried out between April and December 2022. HCP from all over the Netherlands who provided prenatal care in SMA in the last five years were recruited by personal contacts and by snowball sampling. They were approached with a request to participate in a one-to-one interview about the support of self-management. The request also contained the aim of the study and the contact details of the researchers. To achieve data saturation for the codes, we aimed to recruit 15 HCP from as many different provinces of the Netherlands as possible, with an equal distribution from urban and rural areas. The HCP who agreed to participate in an interview, received an e-mail with detailed information about the interview topics and an appointment for the interview, by Microsoft Teams, or face-to-face in a location of the participant's choice.

Interviews were conducted by two midwifery students or by the main researcher. Before the start of the interview, the HCP was asked to sign an informed consent form. In this form, she declared that her participation in the interview was voluntary, that she was informed that she could stop the interview at any time and that she had the right to unilaterally withdraw her data. The HCP was also asked to declare that she was aware of the fact that the interview was recorded and that the data were used only for scientific purposes.

**Fig. 1:**
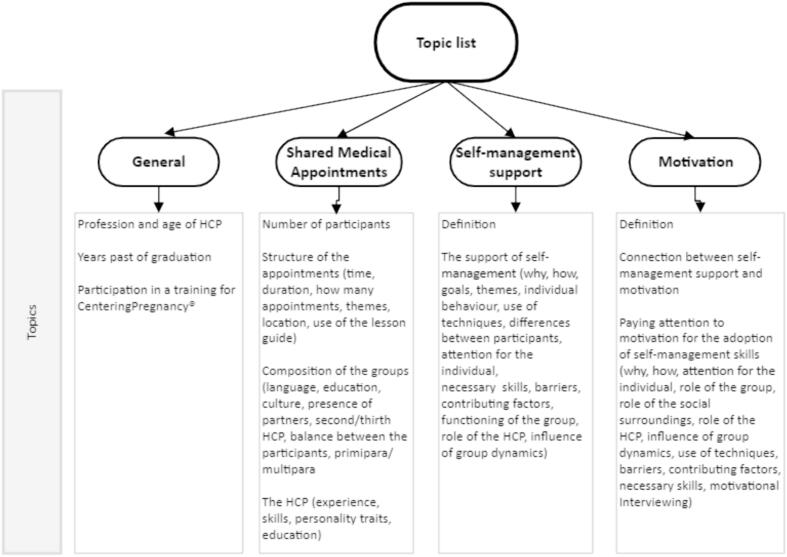
Topic list.

Interviews followed a topic list (Fig. 1) that was the base for open-ended questions as “how do you support self-management” and “which techniques do you use”. This topic list was made by the researchers and based on the research questions, a questionnaire study from Tsiamparlis et al. [39] about the support of self-management by HCP and on an extended literature search (see list of references). Open-ended questions were followed up by clarification questions. In order to ensure rich data, the topic list was critically reviewed by the researchers after each interview and additional topics were added.

Interviews were recorded and transcribed verbatim. Each draft transcript was sent to the interviewee for a member check. After approval of the transcript, the transcript was coded in MAXQDA by two researchers (A.T-W and E.F-DJ), before conducting a new interview. For this coding, thematic analysis was used performing four steps, namely becoming familiar with the data, generating initial codes, forming categories and defining themes [41]. The first and second step of the coding were done by both researchers independently. After completing the second step, the research team discussed about the deviant codes in order to reach consensus about the final codes, categories and themes. Choices were documented in an audit trail.

Related to their voluntary participation in the interview, we have not identified any risk for the HCP. Data were treated in accordance with the Personal Data Protection Act. The Ethical Board of University Medical Center Groningen provided us with a formal declaration (METc 2023/091) that this qualitative study based on interviews with competent individuals does not fall under the scope of the Dutch Act for Medical Research Involving Human Subjects (WMO).

## 3 Results

We conducted 15 in-depth interviews with HCP from 10 (out of 12) Dutch provinces. They all were female, 14 of them were midwives and one was a maternity care assistant (Table 1). All HCP were trained in SMA. The SMA they led, consisted of 6–12 participants and gathered during 9–10 meetings from 1,5–2 h.

**Table 1:** Baseline characteristics of the health care providers providing prenatal care in Shared Medical Appointments (*N* = 15).

Baseline characteristics	N = 15
Profession	
Midwife	14
Maternity care assistant	1
Age in years	
20–40	5
41–70	10
Years past from graduation	
0–10	5
11–20	6
21–30	1
31–40	3
Participation in a training for CenteringPregnancy©	
yes	15
no	0

**Fig. 2:**
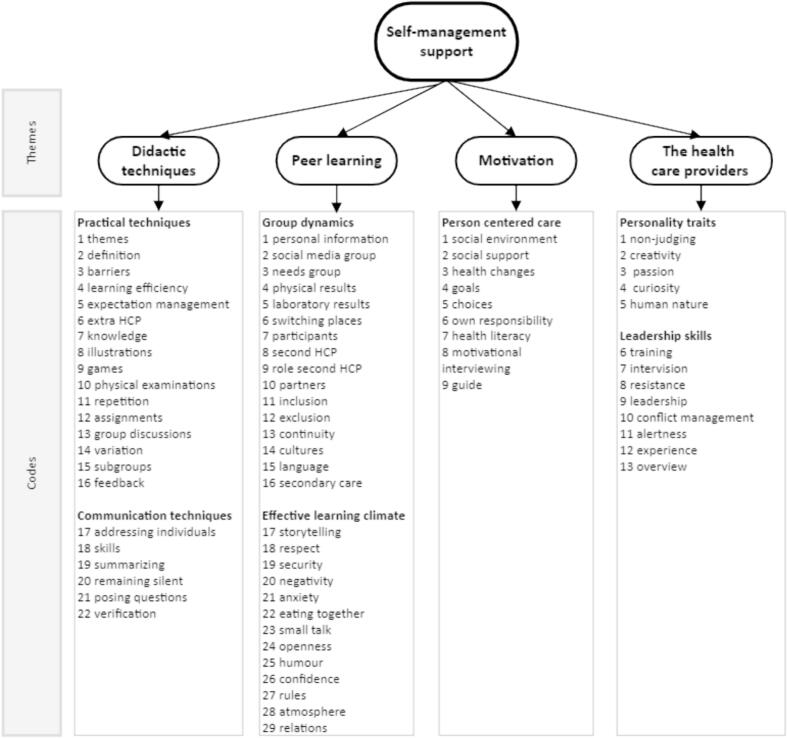
Code tree.

Based on our data analysis, we defined four research themes from the initial codes, namely **didactic techniques, peer learning, motivation and the health care providers** (Fig. 2).

### 3.1 Didactic techniques

Didactic techniques play an important role in the SMA given the fact that for the HCP knowledge acquisition is an important aspect in the support of self-management. Sometimes HCP invite another, specialized HCP for a lecture, but they mostly encourage participants to find the knowledge by themselves. “You don't want to be too dictating […] Not to become a school teacher during CenteringPregnancy@, you are a kind of a supervisor, you try to supervise the process” (R15). “We put it in their own hands, I try less to find solutions for them”(R1). However, they do not always find this an easy job “as they are used to spread information in individual appointments”(R4).

To make participants ready for knowledge acquisition, the HCP use **practical techniques** as games, assignments, illustrations. They also encourage participants to have discussions dividing the group into small working groups. To make sure that participants keep the knowledge in their long-term memory, the HCP make use of variations in techniques and of repetition. As none of the HCP is an experienced teacher, they regularly ask feedback from the participants about their didactic techniques. They ask them for example if they want to discuss supplementary themes, if they like the played games or if they like working in subgroups, changing their techniques according to the feedback of the group. “I always evaluated if I met their expectations and if they had any additions, tips and then I learned from this and used this in future” (R2). As self-assessments are part of the CenteringPregnancy@ program, most of the HCP ask participants to write their own results in their CenteringPregnancy@ guide, “knowing that […] writing the results down, give people self-management […]. They think more about: what were my last results, what is normal and what not?” (R8).

In order to encourage participants to find answers and solutions by themselves, the HCP make use of **communication techniques**. One of these techniques is remaining silent. “I think […] if I give her the answer, I provide bad health care, because I deprive her of thinking about it by herself, I deprive her of the discussions with others, I deprive her of all examples that other people give and that I have never heard of” (R8). A second communication technique they use is posing a lot of questions. “Then someone says, you are not allowed to drink Red Bull. And then we ask, but why not? […] then another participant says, because it contains caffeine. Oh, yeah, that is true, but what else contains caffeine? Oh, yeah, coffee. Are you allowed to drink unlimited coffees?” (R12). The HCP agree that the efficacy of posing questions as a communication technique in the support of self-management is bigger when they pose open questions, but sometimes they find this difficult. “Sometimes it is quite difficult to pose open questions, that I define the answer just by the way I pose the question, that is what I find quite difficult” (R11). A third communication technique the HCP use for involving all participants in group discussions is addressing individuals and ask them to answer questions or to express their opinion. To make clear if an answer given by a participant is correct or not, the HCP summarize the given answer or complete it, using the techniques of summarizing and verification. “If they say very strange things that are false I of course correct the answer and give them the correct information. For the rest, if the group just say correct things, I only confirm their information” (R15).

### 3.2 Peer learning

Learning from peers plays an important role in the support of self-management: “People give each other suggestions. How things can be improved. In this way, the group helps each other with ideas” (R5). The fact that all groups make use of a Social Media group to share information reflects the importance of peer learning for the participants. The HCP actively stimulate peer learning. Based on the Centering guide, they encourage participants to do their own physical check-ups and self-assessments, discussing in the group what are normal values and what not. In this way participants learn to care about their own health at that moment and in the future. “My aim is […] for that blood pressure that you […] know when you have to alert somebody, that you know what are the signals […].” (R8). As all participants of the SMA are obliged to sign a confidentiality form declaring that they keep confident everything they hear and see in the appointments, the HCP feel free to stimulate peer learning by asking participants to voluntarily share and discuss their laboratory results as hemoglobin, blood sugar levels, negative Rhesus factors and ultrasound results in the group. But they are careful to discuss more controversial results. “[…] but I would not mention someone who has a positive HIV test or a positive syphilis test” (R2). The HCP adjust the program of their meetings to the needs of the group. “If I know that I have people with diabetes or a big risk for diabetes in my group, I pay more attention to this theme” (R8).

According to the HCP, peer learning is influenced by **group dynamics**. Prenatal SMA are heterogenous (nulliparas, multiparas, mix of education levels and of cultures) and group composition plays an important role in group dynamics. “Group dynamics varies a lot per group. Highly educated, lowly educated, a former user of drugs, low social class, someone who is familiar with domestic violence” (R10). In order to keep the group in balance, the HCP are constantly aware of group dynamics. “You always have to consider the type of participants and the type of group. In some groups, participants help each other to obtain knowledge and skills, but in some groups they discourage each other. This can be different in every group […]. You should pay attention to this” (R13).

The HCP see the importance of peer learning especially for women from lower socio-economic status, for unemployed women, for single women, for women with few social contacts and for anxious women. “If you are a single mother, this is very good, because you have a source of information and […] this make you more relaxed in pregnancy. You know where to pose your question and they can help you” (R12). The HCP also mention that they sometimes try to persuade women to participate in the SMA. “I want them to experience the feeling of a warm bath with nice women surrounding them.” (R13). On the other hand, the HCP exclude certain persons because they foresee difficulties for group dynamics if they include them. “[…] sometimes I think, I do not want you in a group. Yes, to be honest, I think, she will eat the whole group” (R1). The same difficulties for peer learning, the HCP foresee for participants that do not speak enough Dutch or English, although they suppose that for self-management support participation in a group would be better for these people. “You know, you often make extra time for them in individual appointments […] and the question is if they understand everything there. So the question arises if these persons would not obtain more knowledge the moment they participate in a group” (R8).

A special aspect of group composition is the presence of partners in the SMA. Most HCP allow partners to participate in two meetings (as is established in the CenteringPregnancy@ program), two HCP allow them in all meetings. One HCP who allows partners to participate in all meetings, mentions that she separates the participants and the partners when they speak in the group about domestic violence and about contraception (R10). According to the HCP, group dynamics change positively when partners are present. “When men came to the meetings […] there was more fun I think [..] and sometimes a little bit of showing of. It was a different atmosphere, but a good one, yes” (R2).

As group dynamics influence peer learning, the HCP use some techniques in order to create an **effective learning climate** for peer learning. The first technique they use is the creation of a good atmosphere, for example by promoting small talk. “When you look at the first meeting, people are anxious […]. Then I take care of being present in an obvious way and instead of keeping my mouth shut […] I start a conversation with participants. […] That kind of small tricks to see how I can make them comfortable” (R14). The HCP also contribute to an open atmosphere by sharing personal information, intentionally making themselves peers of the participants. “I think that when you are open yourself and when you dare to be yourself, that this give safety to people, that they think, she dares to say this, so I can do the same” (R1). All HCP provide care to the group together with other HCP who also become peers of the participants. These providers are nurses, midwives, maternity care assistants or midwife's assistants. Eating together and the use of humour are other techniques that the HCP use in creating an effective learning climate. “I always use humour […] for example when I tell them that at a birthday party no one is talking about hemorrhoids, but that many women suffer from them” (R1).

Next to the creation of a good atmosphere, the HCP pay attention to bonding and safety. For this reason they give rules to the group. “That they respect each other, that they let each other finish their sentences, that what is said in the group stays in the group […], no use of mobile phones, just very basic”(R1). “That you really hear the other […] and that she is not judged for her words, that it is safe” (R11). Creating an atmosphere of openness and a feeling of security have positive impact on peer learning and on self-management. “I always […] emphasized that nothing is crazy or weird or strange. If you don't understand it now, just tell it please and maybe we can explain it to each other […] I kept on emphasizing that safety and good atmosphere are important, that stupid questions don't exist, that no answer is wrong […]. And then you see that the group takes responsibility […] and that they all support and help” (R2). On the other hand, the HCP also mention negative effects of an open atmosphere and feelings of bonding and safety. When participants feel secure enough to express negative thoughts, they unintentionally might create anxiety for other participants. “There was […] a client in one of the Centering groups who's fetus had a heart defect. […] And it was quite a severe heart defect. And they were thinking about an abortion. And she shared with the group all her thoughts and doubts about an abortion” (R7). The HCP try to nuance negative feelings and to diminish anxiety in the group. On the other hand, the HCP give space to negative feelings by talking about them: “For example when one of the participants had suddenly an intra-uterine death […] then you have to change the program. […] The group became closer and somewhere they were grateful that their fetus was still alive, but they also experienced a lot of grief […] and who will tell me that this is not going to happen to my fetus. Then we talked together a lot about it and then you hope they will find their trust back”(R2).

### 3.3 Motivation

Despite the fact that in the SMA self-management support is based on peer learning, the HCP mention some elements of person centered care that they pay attention to in motivating participants to adopt self-management skills. One of these elements is goalsetting and the repetition of the goals. “We have these cards with personal goals. […] These assignments really help with self-management”(R12). “[…] which goals do you have to work at? Which goal is difficult for you to achieve? Which goals did you already achieve?” (R15).

Except for goalsetting, the HCP motivate participants to make their own choices and to become responsible for their own health. “I am not going to change them, that is not what I want, that is not my purpose. The aim is that they make changes themselves and that they learn how to do that and what is working for themselves or not” (R8). In motivating participants, the HCP use certain techniques of Motivational Interviewing.

Some HCP, however, find it difficult to motivate participants in themes as individual weight gain and smoking cessation, being afraid of stigmatizing people. When the group want to discuss these themes, the HCP allow this but they try to generalize the discussions and to avoid making them personal. “There is always someone in the group who says, […] you have to stop and that is really better for your child. And then I am really glad […]. I than I can say, we know smoking is one of the most difficult addictions to stop with. Is there someone in the group who ever stopped smoking in her life, or do you know someone who ever stopped smoking and what are your suggestions, what helped you?” (R8). In such delicate questions, the HCP use two techniques, namely keeping it general and using the support of the peers. “If I tell people who smoke, you have to stop smoking, they will not do this, they already know this, because they hear it everywhere. […] Once I had someone in the group who said […] I don't want to talk about smoking. And then I told her, that is difficult, I can't make that promise. […] The only promise I can make you is that you don't have to open your mouth, you don't have to tell that you smoke. […] We discussed smoking and why you shouldn't smoke in pregnancy and tricks for smoking cessation […]. And she didn't speak a lot in that meeting. And in the next meeting […] she told, I stopped smoking. And then I thought, if I had seen her in an individual appointment, she for sure didn't stop smoking, as I was not allowed to discuss the theme. […] that is really nice. […] It is the power of the group” (R8). The HCP also mention support from the peers for other health changes than smoking cessation. “If someone says […] I don't like sports […] and then they make an appointment for walking together” (R14).

### 3.4 The health care providers

The HCP mention that providers need to have certain **personality traits** for the support of self-management in SMA. One of the necessary traits they mention is being open-minded. “That you are an open person, that you don't find anything weird, everything is possible, in that way” (R11). Another trait they need is to feel passion for working with groups and to have interest in human nature. “You must especially be enthusiastic for giving information in this way to a group (R5). The HCP also mention that they must be creative and flexible. “The whole time I am busy thinking of themes, educational activities, making, developing, trying out. To check what helps participants and what not” (R7).

The HCP also mention that they need **leadership skills** in order to be able to create a favorable learning climate. “When you did individual appointments in the appointment room for 100 years and then suddenly you are surrounded by 12 women. Yes, the first time I found it quite enervating” (R13). “That is what we always say, 70% or 80% of group dynamics is in the hand of the health care providers. So, if it does not work, it is in their hands” (R8). As a leader, the HCP are aware that they have to avoid conflicts. “ […] there are tricks to diminish the tension. […] I always keep things very general, I never ask people's preferences. […] And I think that this is one of the most important things that gives security and avoids conflicts” (R8). The HCP also mention that they must be able to manage the group without being too directive. “You can sit there the whole evening without saying very much, because they will talk. Yes, but you have to take care of your management and to achieve the aims you set for the evening” (R3). In order to obtain the necessary skills, the HCP mention that they need more training. “About group dynamics, I always find this a tough part […], we are not educated for this, I find this difficult, from nature I did not get talent for this […] I am good in teaching in old school fashion, but […] I much more like this. […] I can still learn a lot in this” (R11).

## 4 Discussion and conclusion

### 4.1 Discussion

#### 4.1.1 Summary of findings

In SMA, the HCP use different didactic techniques in the support of self-management. They use practical techniques as games, assignments, illustrations and communication techniques as remaining silent, posing questions, summarizing and confirming information. The base of the self-management support is peer learning. This peer learning is influenced by elements of group dynamics as group composition and bonding. For this reason, the HCP are constantly aware of group dynamics and play an active role in the creation of an effective learning climate for peer learning. HCP motivate the participants to adopt self-management skills by peer learning, but they also pay attention to elements of person centered care as goalsetting and empowerment. Being trained in providing care in individual appointments, the HCP mention that they find it quite challenging to work with groups. For an effective support of self-management, they find that HCP need personality traits as extraversion, open-mindedness and passion for working with groups. They also need leadership skills as being able to avoid conflicts, to keep clear overview and to manage the group without being too directive.

#### 4.1.2 Interpretation of findings

By using didactic techniques, by stimulating peer learning and by being alert on group dynamics, the HCP aim to raise participants' awareness for self-management and to create an effective learning climate for the adoption of self-management skills. Peer learning stimulates participants to acquire knowledge with the help of their social surroundings [42], but the HCP also use peer learning as a means of motivation for the adoption of self-management skills.

Next to the didactic concept of peer learning, the HCP use a person centered approach of care in motivating participants to adopt self-management skills for health behaviour changes. They help individuals to set goals and they empower them to make choices. However, they avoid to persuade them and to expose them in the group. Instead, they use their peers to persuade them to adopt skills effectively. By means of peer learning and person-centered care, the HCP in SMA indirectly focus on the three motivation factors of the I-Change model for behavioral changes [22], namely attitude, social influences and self-efficacy. This finding contradicts the findings in the studies from Hughes et al. [24] and Tsiamparlis et al. [39], where health care providers focused on awareness factors and information factors rather than on motivation factors for the adoption of self-management skills. A possible explanation for this contradiction might lie in the perception of the HCP of the concept “motivation”. In the quantitative study of Tsiamparlis et al. [39], the HCP had to choose to which factors they paid the most attention in supporting self-management and they probably considered transferring information and raising awareness the most important factors for motivational support. Even in the present study, the HCP mention that they hesitate to use the word “motivation”, considering the word being part of a paternalistic approach. For this reason, they indirectly try to motivate the participants by raising their self-awareness by knowledge acquisition and self-assessments. This reflects the findings in a study of Armstrong [2], where HCP express their opinion that motivation for health behaviour changes should be intrinsic and that patients need to believe in their own capability to change before they really can change.

By mentioning the fact that they find it difficult to work with groups, the HCP show that they struggle with the balance between an individual, more persuasive approach as they were taught to use in individual appointments and a more neutral approach as they want to use in the SMA. This struggle is also described in a study of Baldwin&Philips [43], where midwives mentioned that they found it a challenge to feel comfortable with “facilitation skills” when they implemented prenatal SMA.

#### 4.1.3 Strengths and limitations

As far as we are aware, we are the first to investigate how HCP use group-based education to support self-management. It is also the first study in which the perception of the HCP of the influence of group dynamics on self-management support and the way that HCP motivate participants for the adoption of self-management skills are examined. For this reason, we think that our findings have added value for patient education. A strength of our study is the fact that we made use of a sampling strategy. By interviewing 15 HCP from 10 different provinces, we got in depth information from as many as possible different perspectives. The fact that we did not get new information in the last two interviews manifests that we reached data saturation for the codes. Another strength is the use of a topic list that is based on former research and on an extensive amount of literature about self-management and motivation, covering the topics needed to answer the research questions. All interviews were recorded and checked by a member check, which raised validity. Besides, the analysis of the data by two researchers independently and the use of thick description raised the trustworthiness of our results. The use of an audit trail made our study more reproducible. A possible limitation of our study might be the fact that the HCP might have given socially desirable answers in order to show that they are good in leading SMA. However, the HCP we interviewed were very open and they also showed critical reflections on their own acts and behaviour, so we have no indication that giving socially desirable answers was a major issue. Another possible limitation of our study can be the fact that HCP that have good insight in educational strategies, group dynamics and self-management support are more motivated to participate in an interview. However, we think that we diminished the risk for this limitation by the fact that all HCP agreed to participate in an interview about SMA before they knew the exact scope of the interview and that none of them withdraw her participation after being informed about the exact scope.

### 4.2 Innovation

The findings of this study give a first insight in the support of self-management in SMA. HCP in all forms of group education can use these insights as well as future HCP. By paying attention to these findings in medical curricula, future HCP may be taught the necessary skills for this kind of patient education. Especially, attention should be given to leadership skills as being able to avoid conflicts, to keep clear overview and to manage the group without being too directive. Future HCP should be taught in the medical schools that the SMA are a good alternative for individual appointments, but they should be aware of the fact that they need insight in didactic techniques, peer learning and group dynamics.

### 4.3 Conclusion

Self-management support in SMA is based on peer-learning and influenced by group dynamics. HCP create an effective learning climate by using practical and communication techniques and they motivate participants for self-management through peer learning and person-centered care. HCP are aware of group dynamics and they need certain personality traits and leadership skills to deal with it.

## CRediT authorship contribution statement

**Anna H.C. Tsiamparlis-Wildeboer:** Writing – original draft, Visualization, Software, Resources, Project administration, Methodology, Investigation, Formal analysis, Data curation, Conceptualization. **Esther I. Feijen-De Jong:** Writing – review & editing, Methodology, Formal analysis, Data curation, Conceptualization. **Elke Tichelman:** Writing – review & editing, Visualization, Software. **Ank de Jonge:** Writing – review & editing, Supervision. **Fedde Scheele:** Writing – review & editing, Supervision, Methodology, Conceptualization.

## Declaration of competing interest

None.

We confirm all personal identifiers have been removed or disguised so the persons described are not identifiable and cannot be identified through the details of the story.
